# Activities and Molecular Mechanisms of Diterpenes, Diterpenoids, and Their Derivatives in Rheumatoid Arthritis

**DOI:** 10.1155/2022/4787643

**Published:** 2022-03-25

**Authors:** Muhammad Torequl Islam, Cristina Quispe, Jesús Herrera-Bravo, Md. Mizanur Rahaman, Rajib Hossain, Chandan Sarkar, Md Abdur Raihan, Md. Mashrur Chowdhury, Shaikh Jamal Uddin, Jamil A. Shilpi, João Marcelo de Castro e Sousa, Ana Amélia de Carvalho Melo-Cavalcante, Mohammad S. Mubarak, Javad Sharifi-Rad, Daniela Calina

**Affiliations:** ^1^Department of Pharmacy, Life Science Faculty, Bangabandhu Sheikh Mujibur Rahman Science and Technology University, Gopalganj 8100, Bangladesh; ^2^Facultad de Ciencias de la Salud, Universidad Arturo Prat, Avda. Arturo Prat 2120, Iquique 1110939, Chile; ^3^Departamento de Ciencias Básicas, Facultad de Ciencias, Universidad Santo Tomas, Santiago, Chile; ^4^Center of Molecular Biology and Pharmacogenetics, Scientific and Technological Bioresource Nucleus, Universidad de La Frontera, Temuco 4811230, Chile; ^5^Department of Pharmacy, Southern University Bangladesh, Arefin Nagar 4210, Chattagram, Bangladesh; ^6^Pharmacy Discipline, Khulna University, Khulna 9208, Bangladesh; ^7^Postgraduate Program in Pharmaceutical Sciences, Federal University of Piaui, Teresina 64049-550, Brazil; ^8^Department of Chemistry, The University of Jordan, Amman 11942, Jordan; ^9^Facultad de Medicina, Universidad del Azuay, Cuenca, Ecuador; ^10^Department of Clinical Pharmacy, University of Medicine and Pharmacy of Craiova, 200349 Craiova, Romania

## Abstract

Diterpenes and their derivatives have many biological activities, including anti-inflammatory and immunomodulatory effects. To date, several diterpenes, diterpenoids, and their laboratory-derived products have been demonstrated for antiarthritic activities. This study summarizes the literature about diterpenes and their derivatives acting against rheumatoid arthritis (RA) depending on the database reports until 31 August 2021. For this, we have conducted an extensive search in databases such as PubMed, Science Direct, Google Scholar, and Clinicaltrials.gov using specific relevant keywords. The search yielded 2708 published records, among which 48 have been included in this study. The findings offer several potential diterpenes and their derivatives as anti-RA in various test models. Among the diterpenes and their derivatives, andrographolide, triptolide, and tanshinone IIA have been found to exhibit anti-RA activity through diverse pathways. In addition, some important derivatives of triptolide and tanshinone IIA have also been shown to have anti-RA effects. Overall, findings suggest that these substances could reduce arthritis score, downregulate oxidative, proinflammatory, and inflammatory biomarkers, modulate various arthritis pathways, and improve joint destruction and clinical arthritic conditions, signs, symptoms, and physical functions in humans and numerous experimental animals, mainly through cytokine and chemokine as well as several physiological protein interaction pathways. Taken all together, diterpenes, diterpenoids, and their derivatives may be promising tools for RA management.

## 1. Introduction

Arthritis is a long-term musculoskeletal illness marked by inflammation of the joints. Rheumatoid arthritis (RA) is one of the most common kinds of arthritis [1]. It is a long-term condition marked by inflammatory synovitis. Joint asymmetry and invasive inflammation are common symptoms of RA, which can lead to joint deformity, dysfunction, and even loss of function. Adults in rich countries have a prevalence of 0.5–1.0%, with 5–50 new cases per 100,000 persons each year. Women and the elderly, on the other hand, are the ones who suffer the most [2]. Although the exact origin of RA is unknown, medicinal therapy is a common and effective treatment option for RA patients.

Treatments for RA include nonsteroidal anti-inflammatory medications (NSAIDs), corticosteroids, disease-modifying antirheumatic medicines (DMARDs), and biological response modifiers [3]. All of these anti-RA drugs, unfortunately, have numerous negative effects. NSAIDs may endanger patients' lives by increasing the risk of upper gastrointestinal (GI) haemorrhage, liver, and kidney damage [4, 5]. Furthermore, headaches, cognitive impairments, and allergic reactions are common reasons for patients to stop taking NSAIDs, limiting their usage. Infection, hypersplenism, hypertension, osteoporosis, and fractures are all possible side effects of long-term corticosteroid usage [6, 7]. Vomiting, diarrhea, rashes, low white blood cell (WBC) counts, and impaired liver and renal function are also side effects of DMARDs [8, 9].

Biological therapies with high pharmacological selectivity and fewer side effects provide novel RA treatment alternatives [10]. Regrettably, these are pricey. As a result, many patients may be unable to afford these drugs [11]. As a result, it is critical to seek out treatments that have a positive therapeutic benefit, few side effects, and are affordable. Many ailments are treated according to conventional medical principles. Many major studies on therapeutic items with natural origins have been conducted by modern scientists.

Plants or their derivatives, marine items, and so forth are examples. These natural items have been discovered as a promising treatment option for RA [12]. Aside from that, several conventional pharmaceutical formulae for RA care have been difficult. Two significant features of this method are the use of nutraceuticals and polyherbal approaches [13, 14]. However, when these preparations are used in combination with other drugs, they may cause health problems. As a consequence, researchers devised a novel strategy for extracting active chemicals from some of these things. Terpenes, flavonoids, catechins, quinones, alkaloids, anthocyanins, and anthoxanthins are just a few of the plant-derived phytochemicals that can alter T cell development, inflammatory signaling pathways, and synoviocyte death. As a result, they can be utilized to treat rheumatoid arthritis [15].

Diterpenes are a diverse group of structurally diverse natural chemicals abundant in nature [16, 17]. These are C20 compounds containing four isoprene (C_5_H_8_) units that may be found in both terrestrial and marine settings in plants, fungi, bacteria, and animals [18, 19]. Several diterpenes are potential pharmaceutical candidates due to their exceptional pharmacological effect [20–23]. Some diterpenes are considered to be the defining traits of a genus, making them taxonomically significant [24].

Natural diterpenoid compounds come in a wide range of chemical forms and contain many medicinal and economically relevant molecules. All diterpenoids are made from the same substrate, (E, E, E)-geranylgeranyl diphosphate, which is then cyclized into one of the multiple scaffolds by a diterpene synthase [25]. Secondary metabolites with 20 carbon atoms result from the condensation of four isoprene units.

Diterpenoids are divided into approximately 45 distinct categories, and they are also present in marine animals, where their skeletons are fascinating [26]. Based on their skeletal nucleus, diterpenes are classified as linear, bicyclic, tricyclic, tetracyclic, pentacyclic, or macrocyclic. They are usually found in nature polyoxygenated, with keto and hydroxyl groups that are commonly esterified by tiny aliphatic or aromatic acids [27].

Diterpenoids have a variety of biological functions, including antioxidant [23, 28], anti-inflammatory [29, 30], and immune-modulatory action [31]. Given the significance, the goal of this study is to outline the effects of diterpenes, diterpenoids, and their derivatives on RA based on current understanding.

## 2. Review Methodology

A search with the keywords “Diterpene AND Rheumatoid arthritis,” “Diterpenoid AND Rheumatoid arthritis,” “Diterpene AND Arthritis,” “Diterpenoid AND Arthritis,” “Diterpene derivative AND Rheumatoid arthritis,” and “Diterpene derivative AND Arthritis” was conducted in the PubMed, Science Direct, Google Scholar, and Clinicaltrials.gov databases. A total of 2708 records were found. After screening, among them, this study used 48 published records that are related to its aim.

This study includes only the records of having antiarthritic or anti-RA along with anti-inflammatory activities of the diterpenes, diterpenoids, and their derivatives obtained from various sources (e.g., medicinal plants and marine origins) on various test systems (e.g., humans), laboratory animals (e.g., mice, rats, and rabbits), and their derivatives (e.g., cells, tissues or organs).

Most of the diterpenes, diterpenoids, and their derivatives have antioxidant and anti-inflammatory properties, and for this reason, this study does not include them. Reports on crude extracts or fractions without chemical characteristics having antiarthritic or anti-RA effects were also excluded in this study.

This study mainly focuses on the anti-RA activities of diterpenes, diterpenoids, and their derivatives. However, it will also focus on the antiarthritic effects of these substances based on updated database records (till 31 August 2021).

## 3. Physiopathology in RA: A Brief Overview

Since cytokines are directly involved in RA pathogenesis, they have been intensively explored and examined as potential RA targets. Cytokines can be classified as pro or anti-inflammatory cytokines according to their antigen response activities. TNF-*α*, interleukins (ILs) (e.g., IL-1*β*, -6, -7, -15, -17, -18, and -23), interferon-gamma (IFN-*γ*), and granulocyte-macrophage colony-stimulating factor (GM-CSF) have all been found to limit inflammation in the progression of RA. In the synovium, synovial fluid, serum, and peripheral blood of RA patients, these cytokines were detected in high amounts [32–38].

T cell trafficking and proinflammatory cytokines such as TNF-*α*, IL-1*β*, IL-6, and MMPs are reduced when IL-7 is blocked, which lowers joint inflammation [39]. The major cause of IL-23-induced synovial inflammation (RORs) is the activation of Janus kinase (JAK)/signal transducer and activator of transcription (STAT), tyrosine kinase 2, NF-*κ*B, and retinoic acid receptor-related orphan receptors [40].

Macrophages can produce a variety of cytokines such as TNF-*α*, IL-1*β*, -6, -7, -15, -18, and -23. In this regard, TNF-*α* may stimulate fibroblast-like synoviocytes (FLS) and synovial cell proliferation through nuclear factor kappa-B (NF-*κ*B) and extracellular regulated protein kinases (ERK)-1/2-E26 transformation-specific (ETS)-1 regulatory pathways [37]. Consequently, several inflammatory mediators such as IL-6 and matrix metalloproteinases (MMP), MMP-1 and MMP-3, are secreted and increase inflammation [41].

Small molecular metabolites such as PGs, lipoxins (LXs), platelet-activating factor (PAF) and leukotrienes (LTs), nitric oxide (NO), and ROS play important roles in the physiopathology of RA [42]. PG expressions such as PGD2, PGE2, PGF2a, PGI2, PGJ2, and TXA2 are aberrant in RA [42]. LXs derived from arachidonic acids, such as LXA4 and LXB4, possess anti-inflammatory properties. LXA4 can reduce memory B cell response in RA patients' synovial tissues by engaging the lipoxin A4 receptor (ALX)/formyl peptide receptor-2 (FPR-2) and, therefore, reduce inflammation [43, 44]. Circulating platelet activation affects leukocyte activity and contributes to inflammation development in RA patients [45]. TNF-*α*-regulated pathways are known to control PAF, and TNF-*α* antagonists decrease platelet activation in active RA [46].

Chemokines have a role in the underlying pathophysiology of RA by attracting leukocytes and influencing angiogenesis. Published research indicated that XC chemokines and their receptors (such as XCL1 and XCR1) and CX3C chemokines and their receptors (e.g., CX3CL1 and CX3CR1) are upregulated in RA patients' mononuclear cells (MNCs) and FLS, respectively [47, 48]. Numerous inflammatory chemokines are mostly generated in the joints of RA patients by synovial macrophages and FLS, while CX3CL1 is produced by synovial endothelial cells. The chemokines XC and CX3C are linked to the recruitment of T lymphocytes and synovial fibroblasts. Furthermore, CX3CL1 and XCL1 stimulate the migration of monocytes and subchondral mesenchymal progenitor cells into the RA synovium, respectively [49]. CC chemokines including CCL2–5, CCL7, CCL13, CCL14, CCL16, CCL18–21, and CCL-25 are differentially expressed in RA plasma and synovium [50]. An upregulated CC chemokine CCL5 is significantly correlated with swollen joints, erythrocyte sedimentation rate (ESR), and c-reactive protein (CRP) in RA patients [51]. On the other hand, CXC chemokines, such as CXCL1, CXCL2, CXCL5, CXCL8, CXCR1, and CXCR2, are generally involved in neutrophil chemotaxis [52]. The chemokine CXCL10 promotes effector T cells into the joint [53].

The expression of peroxisome proliferator-activated receptor-gamma (PPAR*γ* or PPARG) in human monocytes/MDMs may be an indication of disease activity and treatment effectiveness in RA. Several studies have shown that key cell types in the joints [54, 55] express PPAR*γ* at both the mRNA and protein levels.

Long noncoding RNAs (lncRNAs) are more than 200 nucleotides in length and are extensively expressed in many organs of the human body. Several researchers have shown that lncRNA could be used to diagnose RA [56, 57].

In RA patients with active synovitis, osteoprotegerin (OPG) expression on macrophage type synovial lining cells and also endothelial cells is low. As a result, addressing OPG expression in RA patients' inflamed joints may be an essential approach for the treatment of RA in humans [58]. The RANKL/OPG pathway is the connecting factor between bone production and bone resorption in the complicated system of bone remodeling. RANKL promotes the activation and differentiation of preosteoclasts and mature osteoclasts by binding to their receptors (RANK).

Certain hormones, growth factors, and cytokines affect the synthesis of RANKL and OPG by osteoblasts in various ways. Thus, the level of proliferation and activity of osteoclasts are determined by the balance of RANKL and OPG. Bone erosions in RA are caused by osteoclastic bone resorption in synovitis sites, in which RANKL expression is also observed [59].

Currently, available anti-RA agents focus on targeting cytokines, chemokines, and various physiological proteins in humans. Adalimumab is an anti-RA medication that prevents TNF and its receptors from binding, thus lowering cytokines (e.g., MMP-1 and MMP-3)-mediated inflammatory mechanisms and cartilage and bone degradation [41]. On the other hand, (5R)-5-hydroxytriptolide can systemically affect the FLS and, in particular, in the process of immune-related processes at 100 nM concentration through a genome-wide microarray assay in RA patients [60]. IL-1 activates the extracellular signal-regulated kinase (ERK), c-Jun N-terminal kinase (JNK), apetala (AP)-1, and NF-B activating pathways, which stimulate MMP production and leukocyte adhesion to RA FLS [61], whereas oridonin (2–10 M for 24–72 h) suppress RA FLS proliferation in RA [62]. In RA FLS, (5R)-5-hydroxytriptolide (50 and 100 nM) reduced proliferation and invasion, as well as cytokine production (MMP-3, IL-1, and -6) [63]. By stimulating the synthesis of MMPs and NF-*κ*B ligand (RANKL) receptors, IL-6 promotes bone resorption and cartilage degradation [64, 65].

**Figure 1:**
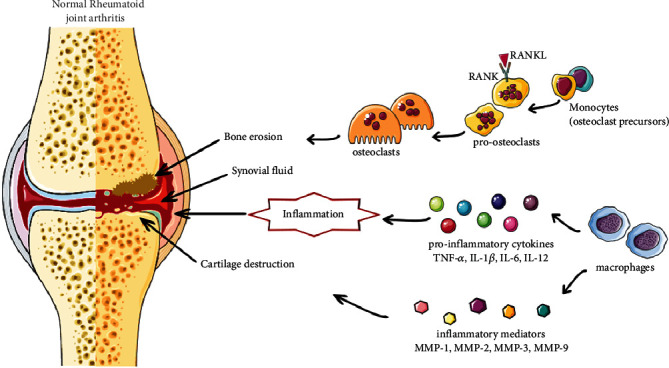
Diagram with the most representative mechanisms of RA pathogenesis.

NSAIDs work by reducing the enzymatic activity of the cyclooxygenase (COX) enzymes, which are involved in the production of prostaglandins (PGs). NSAIDs inhibit COX-2, which limits PG synthesis at sites of inflammation; however, inhibiting COX-1 in other tissues (e.g., platelets and mucosa) results in classic NSAID side effects such as bleeding and GI ulcers [66]. Summarized scheme of the physiopathology of RA is shown in Figure 1.

## 4. Anti-RA Activities of Diterpenoids: Actions and Molecular Mechanisms

Diterpenoids are the most prominent source of anti-RA agents with potential pharmacological effects.

### 4.1. Cytokine Targeting Diterpenes and Their Derivatives

A recent study has been claimed that diterpenes isolated from *Caesalpinia minax* (Hance) substantially reduced the change in paw swelling perimeter, arthritic score, and increased bodyweight loss in vivo study [67]. Furthermore, the primary components of the extract were 14 cassane derivatives, such as caesalpins A–H, caesalminaxin A–L, and others, which exhibit a promising effect on the expression of mRNA of the cytokines IL-1*β* and IL-6 and TNF-*α* generated by macrophage cells. Moreover, some other diterpenoids (rhodojaponin III, rhodojaponin VI, 2-O-methylrhodojaponin, and 5′-*β*-D-glucopyranosyloxyjasmonic acid) in *Rhododendron molle* fruits at 0.6 mg/kg dose dramatically reduced RA symptoms in CIA rats [68] by strongly preventing aberrant T and B lymphocyte proliferation and substantially decreased levels of the proinflammatory cytokines IL-1*β* and IL-6, as well as TNF-*α*. It has been seen that (5R)-5-hydroxytriptolide can inhibit IL-1*β*, IL-6, and IL-21 secretion and elevated IL-10 secretion in peripheral blood and synovial fluid of RA patients [69]. Andrographolide in bone marrow macrophages cells and mice inhibited RANKL-stimulated osteoclastogenesis via downregulating NF-*κ*B and ERK/MAPK expression, thereby averting bone loss [70]. Cryptotanshinone at 6 and 18 mg/kg (p.o., for 16 days) in type II collagen-induced arthritis in female Wistar rats, and 5 and 20 *μ*M concentration inhibits the degradation of NF-*κ*B (I*κ*B)-*α* blocker [71].

Research findings indicated that triptolide (1–4 nM/L) inhibited RANKL-induced NF-*κ*B activation and RANKL and tumor cell-induced osteoclastogenesis [72]. Additionally, a derivative of triptolide (5R)-5-hydroxytriptolide downregulated the expression of (p)-I*κ*B, a major regulator of the RANKL-signaling pathway in RA patients' peripheral blood and synovial fluid [69]. Another study suggested that triptolide (2.5–40 nM) enhanced the inhibitory effects of TREGS on osteoclast differentiation and bone resorption through an increase in the secretion of IL-10 and transforming growth factor-beta 1 (TGF-*β*1) in mice bone marrow macrophages [73].

Table 1 provides the list of various diterpenes, diterpenoids, and their derivatives, which act in various RA models.

Triptolide in collagen-induced arthritis rats significantly inhibited triggering receptors expressed on myeloid cells (TREM)-1 mRNA and DAP12 mRNA expression and activation of JAK2 and STAT3 in the ankles of test animals and in LPS-stimulated U937 cells [82].

In MH7A cells, retinoic acid-platinum (II) complex (0.25–12 *μ*M) downregulated the activation of the MEK/NF-*κ*B pathway [90], whereas sclareol exhibited anti-RA potential in collagen-induced arthritis in vivo and in vitro models [74]. Furthermore, through inhibiting NF-B translocation and MAPK pathway activation, sclareol inhibited the IL-1*β*-induced production of TNF-*α*, MMP-1, and IL-6. Furthermore, sclareol at doses of 5 and 10 mg/kg (i.p.) reduced the number of Th17 cells in mice and improved edema and bone erosion.

In a cytokine-stimulated expression of the major cartilage damaging proteases, MMP-3, MMP-13, and ADAMTS-4 in human and bovine chondrocytes, SW1353 cells, and synovial fibroblasts, triptolide inhibited cytokine-induced MMP-3 and MMP-13 gene expression in primary human OA chondrocytes, bovine chondrocytes, SW1353 cells, and human syn [80]. It also prevented MMP-13 production by IL-1 in human and bovine cartilage explants and IL-1, IL-17, and TNF-*α* induced expression of ADAMTS-4 in bovine chondrocytes.

Triptolide inhibited the IL-1-induced phosphorylation of ERK, p38, and JNK at protein levels in bovine type II collagen-induced arthritis DA rats treated with 11–45 g/kg/day (i.g.) for 28 days and significantly decreased the expression of angiogenic activators such as TNF-*α*, IL-17, vascular endothelial growth factor (VEGF), VEGF receptor (VEGFR), and Ang-1 [81]. Triptolide also lowered the production of TNF-*α*, IL-1*β*, and IL-6 in blood and joints of collagen-induced arthritic rats [82].

Similarly, Kirenol, isolated from *Herba siegesbeckiae*, at 100–200 *μ*g/mL inhibited the migration, invasion, and proinflammatory IL-6 secretion in RA-associated synovial fibroblasts [87]. Moreover, it inhibited the production of proinflammatory cytokines (e.g., IL-6) and synovium hyperplasia and cartilage erosion in a dose-dependent manner in collagen-induced arthritis male DBA/1 mice, whereas tanshinone IIA suppressed IL-6 and TNF-*α* expression and release in neutrophils and promoted neutrophil apoptosis in adjuvant-induced arthritis in female C57BL/6 mice [94].

Phlomisoside F (5, 10, and 20 mg/kg, p.o., for 28 days) inhibited the expression of TNF-*α*, IL-1*β*, IL-6, COX-2, and 5-lipoxygenase (5-LOX), and increased the expression of IL-10 in complete Freund's adjuvant-induced arthritis male Wistar rats [94]. 11-epi-Sinulariolide acetate (9 mg/kg, s.c., once every 2 days from day 7 to day 28 postimmunization) reduced the expression of cathepsin K, MMP-9, TRAP, and TNF-*α* in ankle tissues in adjuvant-induced RA in female Lewis rats [89].

Research findings indicated that retinoic acid-Pt (II) complex (2 and 5 mg/kg, i.g.) drastically decreased IL-1*β*, IL-6, IL-8, MMP-1, and MMP-13 levels in synovial fluid dose-dependently in Sprague-Dawley rats [90]. It also significantly inhibited the expression of iNOS and COX-2 mRNA proteins in RA rats. Furthermore, retinoic acid-platinum (II) complex (0.25–12 *μ*M) reduced TNF-*α*-induced proliferation in a concentration-dependent manner in MH7A cells.

In collagen II-induced arthritis male DBA/1J mice, ginkgolide B (10, 20, and 40 *μ*M, i.p., for 43 days) decreased the serum levels of IL-1*β*, IL-6, TNF-*α*, MMP-3, and MMP-13 and increased the anti-inflammatory cytokine IL-10 [99]. The synovial production of monocyte chemoattractant protein-1 (MCP-1) may be crucial in the recruitment of mononuclear phagocytes during RA inflammation [100]. Xie et al. [99] demonstrated that ginkgolide B significantly decreases the serum levels of chemokine MCP-1 in arthritis animals.

In animal studies, WB2086, a human PAF receptor antagonist, reduces PAF-induced platelet aggregation [101]. Findings showed that sclareol exhibits significant anti-inflammatory effects in experimental animals; it inhibits NO production and upregulates inducible nitric oxide synthase (iNOS) and COX-2 expression in lipopolysaccharide (LPS)-stimulated macrophages [102]. It also decreased paw edema and neutrophil infiltration in the *λ* carrageenan-induced paw edema animal model. On the other hand, aphamines A–C isolated from *Aphanamixis polystachya* exhibited inhibitory effects on NO production (IC_50_: 6.71–15.36 *μ*mol/L) and reduced the expression of iNOS in LPS-induced RAW 264.7 macrophages [103].

Serralabdanes A–E isolated from the whole plant of *Chloranthus serratus* also showed inhibitory effects on LPS-induced NO production in RAW264.7 cells [104]. Other compounds such as tripterycoside A–C, 11-O-*β*-d-glucopyranosyl-neotritophenolide, and wilfordoside A at 10 *μ*M exerted substantial inhibition of IL-1*β* secretion in LPS-induced rat primary synovial fibroblasts [105]. Similarly, researchers found that secoferruginol isolated from the heartwood of *Cryptomeria japonica* modulates human DC function in a fashion that favors Th2 cell polarization [106], whereas songorine, a C_20_ diterpenoid alkaloid and 12-keto analogue of napelline, isolated from *Aconitum soongaricum*, exhibited anti-inflammatory and antiarthritis activities [107].

Some of the important proteins involved in RA include JAK, p38 mitogen-activated protein kinase (MAPK), extracellular receptor kinase (ERK), JNK, IL-1 receptor-associated kinase (IRAK)-4, MMPs, toll-like receptor 4 (TLR-4), G protein-coupled receptor kinase (GRK)-2, Bruton's tyrosine kinase (BTK), CD3, CD11a, CD19, CD20, and CD80. JAK is a component of the JAK/STAT signaling system, which is constantly active, resulting in increased levels of MMPs and apoptotic chondrocytes in RA synovial joints [108]. Published research showed that excavatolide B (2.5 and 5 mg/kg, s.c.) in adjuvant (AIA) and type II collagen-induced arthritis in rats attenuate the protein expression of CD11b and nuclear factor of activated T cells 1 (NFATc1) in ankle tissues [75]. Giannelli et al. [109] reported that evaluation of synovial fluid concentrations of TIMPs (e.g., TIMP-1 and TIMP-2) is more reliable than that determined in serum when remodeling cartilage ECM proteins, besides MMPs evaluated. These researchers suggested that both TIMPs and MMP inhibitors might be a potential target for novel RA treatments administered directly into the joint area. In this respect, triptolide (10, 30, and 50 nM) in RA FLS from 7 RA patients reduced the TNF-*α*-induced expression of phosphorylated JNK [83]. Additionally, it has been demonstrated that a JNK-specific inhibitor reduces the migration and invasion of RA FLS.

Research by Zhong and colleagues showed that sclareol exhibits antiosteoarthritic properties in IL-1*β*-induced rabbit chondrocytes and a rabbit model of osteoarthritis induced by ACLT [110]. Sclareol also inhibited MMP, iNOS, and COX-2 expression and increased TIMP-1 expression and ameliorated cartilage degradation in the test systems. Similarly, 11-epi-sinulariolide acetate significantly inhibited the expression of the proinflammatory proteins iNOS and COX-2 in LPS-stimulated murine macrophages [89]. Moreover, oridonin (2–10 *μ*g/mL for 24–72 h) increased apoptosis, protein levels of Bax, and cleaved caspase-3 in RA FLS. However, it significantly decreased IL-1*β* levels in the test system [52]. Meanwhile, excavatolide B at 10 *μ*M inhibited multinucleated cell and actin ring formation and also tartrate-resistant acid phosphatase (TRAP or TRAPase), MMP-9, and cathepsin K expression in LPS-stimulated RAW 264.7 cells [75].

**Figure 2:**
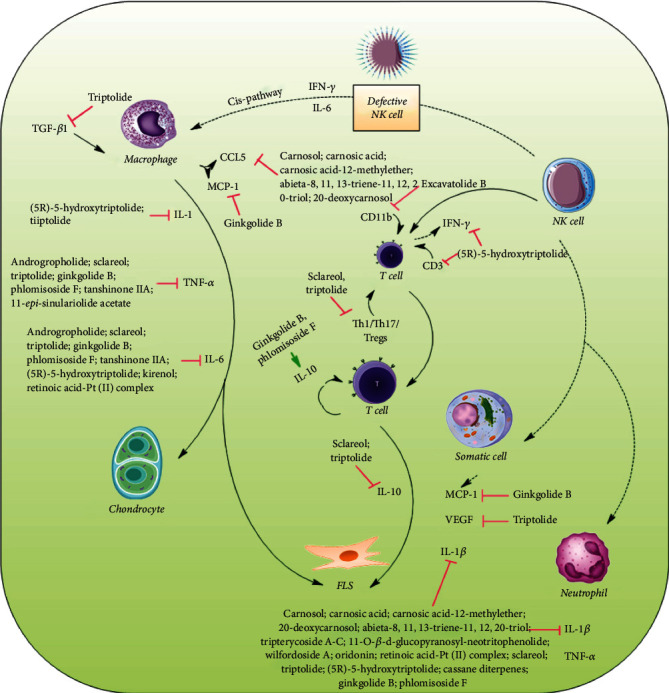
Diterpenes and their derivatives targeting cytokines in RA.

Researchers also demonstrated that the soft coral-derived diterpene at 2.5 and 5 mg/kg (s.c.) significantly attenuated the characteristics of RA, improved histopathological features, decreased the number of TRAP-positive multinucleated cells, and attenuated the protein expression of cathepsin K, MMP-2, and MMP-9 in ankle tissues as well as the level of IL-17A and macrophage colony-stimulating factor in adjuvant (AIA) and type II collagen-induced arthritis in rats.  Figure 2 shows the possible mechanisms of diterpenes and their derivatives targeting cytokines.

### 4.2. Chemokines Targeting Diterpenes and Their Derivatives

A clinical trial of E6011 (an anti-CX3CL1 mAb) is currently underway, and it has been shown to have a potential function in active RA patients [111]. In LPS-induced FLS, ginkgolide B (5–80 *μ*M) remarkably inhibited RA FLS viability in a concentration-dependent fashion. It also reduced the apoptotic ratio and enhanced the expression of cleaved caspase-3 and Bax. Furthermore, it reduced Bcl-2 expression in RA FLS, decreased the development of inflammation by regulating inflammatory cytokine secretion and MMP gene expression, and reduced expression levels of Wnt5a, (p)-JNK, and p-P65 in synovial tissues and RA FLS [88].

Other diterpenes, diterpenoids, or their derivatives that inhibit RA FLS include triptolide [79, 83], tanshinone IIA [93], (5R)-5-hydroxytriptolide [60], Kirenol [87], oridonin [52], and triptolide (TP) loaded with miR-30-5p inhibitor [84].

Findings showed that carnosol, carnosic acid, carnosic acid-12-methyl ether, 20-deoxocarnosol, and abieta-8,11,13-triene-11,12,20-triol significantly blunt gene expression levels of iNOS, cytokines/interleukins (IL-1*α*, IL-6), and chemokines including CCL5/RANTES, CXCL10/IP-10 in murine macrophages (RAW264.7 cells), and human chondrocytes [112].

**Figure 3:**
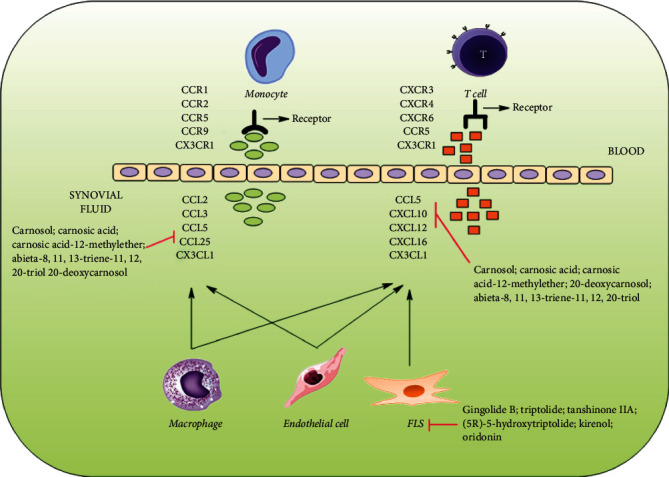
Diterpenes and their derivatives targeting chemokines in RA.

Figure 3 shows the possible mechanisms of diterpenes and their derivatives targeting chemokines.

### 4.3. Diterpenes and Their Derivatives Acting on Other Proteins

Triptolide (0.01–10 *μ*M) downregulated PPAR-*γ* activation and induced DNA fragmentation in RSF in rheumatoid synovial fibroblasts from RA patients [79]. It also decreased arthritis scores and significantly reduced capillaries, small, medium, and large vessel density in the synovial membrane tissues of inflamed joints in bovine type II collagen-induced arthritis DA rats [81]. Moreover, triptolide inhibited Matrigel-induced cell adhesion of HFLS-RA, and HUVEC as well as disrupted tube formation of HUVEC on Matrigel, and suppressed the VEGF-induced chemotactic migration of HFLS-RA and HUVEC, respectively, in arthritis rats.

(5R)-5-Hydroxytriptolide increased the rate of osteoprotegerin (OPG) expression in CD3^+^ T leukomonocytes in peripheral blood and the ratio of OPG/RANKL in both peripheral blood and synovial fluid in peripheral blood and synovial fluid of RA patients [69]. It also inhibited IL-23 secretion in the supernatants of PBMCs and SFMCs in peripheral blood and synovial fluid of RA patients [69]. It additionally prevented collagen-induced arthritis via inhibiting OPG/RANK/RANKL signaling in osteoclastogenesis and IFN-*γ* signaling in T cells [113, 114]. Recently, Zhou et al. [63] demonstrated that it exerts an anti-RA effect through the WAKMAR2/miR-4478/E2F1/p53 dependent pathway in RA FLS. MEG3 lncRNA overexpression reduces inflammation by affecting the AKT/mTOR signaling pathway [115].

Tanshinone IIA (1–80 *μ*M) exerted cytotoxically apoptosis effects through upregulating lncRNA GAS5, possibly with an increase in cleaved caspase-3/9 expression and inhibiting the PI3K/AKT signaling pathway in FLS from RA patients [93]. In addition, numerous studies indicated that PGs play an important role in physiological immune responses and in pathological diseases related to inflammation and tissue damage.

In murine macrophages (RAW264.7 cells) and human chondrocytes, carnosol, carnosic acid, carnosic acid-12-methyl ether, 20-deoxocarnosol, and abieta-8,11,13-triene-11,12,20-triol reduced NO and PGE_2_ production in a concentration-dependent manner. They also significantly reduced iNOS and cytokine (IL-1*α* and IL-6) gene expression levels in the test systems [112]. Additionally, these substances altered the expression of catabolic and anabolic genes in the chondrosarcoma cell line SW1353 and primary human chondrocytes, stimulated by IL-1*β*, where catabolic genes such as MMP-13 and ADAM metallopeptidase with thrombospondin type 1 motif 4 (ADAMTS-4) that contribute to cartilage erosion were downregulated, whereas anabolic gene expression, particularly Col2A1 and aggrecan, was moved towards prepathophysiological equilibrium. Furthermore, carnosol exhibited the greatest overall impact on inflammatory mediators as well as macrophage and chondrocyte gene expression. It significantly inhibited IL-1*β*-induced nuclear translocation of NF-*κ*B-p65, suggesting that it is primarily regulated through the NF-*κ*B signaling pathway. Lobolide, a cembrane diterpene, also acts through the NF-*κ*B signaling pathway [116]. Moreover, andrographolide attenuated mouse cortical chemokine levels from the CC and CXC subfamilies in LPS-induced chemokine upregulation in a mouse model [117].

Table 2 provides the list of diterpenes, diterpenoids, and their derivatives that interact with various proteins involved in inflammatory and RA processes.

### 4.4. Miscellaneous Pathways in RA Treatment

In type II collagen-induced arthritis in rats, triptolide (0.1 mg/kg, p.o., for 28 days) significantly delayed the onset of arthritis. In addition, the arthritis incidence, clinical arthritis severity score, histopathological arthritis severity score, and in vivo cell-mediated immunity to collagen were all reduced [78].

In bovine collagen type II and complete Freund's adjuvant-induced arthritis in DBA/1 mice, cryptotanshinone (20 and 60 mg/kg, p.o., for 6 weeks) ameliorated the inflammation and joint destruction [98]. It also suppressed p300-mediated STAT3 acetylation in test animals. Similarly, carnosic acid at 30 and 60 mg/kg (i.p., 4 weeks) in collagen-induced arthritis in male C57BL/KsJ-db/db mice and at 10 or 20 *μ*M in mouse bone marrow cells reduced osteoclast formation and bone loss through suppression of inflammation by regulating the ROS-dependent p38 pathway [99]. On the other hand, xylopic acid nanoformulation showed anti-inflammatory and anti-RA effects in RAW 264.7 cells and complete Freund's adjuvant-induced arthritis in male Sprague-Dawley rats [97].

Research findings showed that 7*β*-hydroxycalcaratarin A, a labdane-type diterpenoid derived from *Hedychium coronarium*, inhibits superoxide anion generation by human neutrophils in response to formyl-L-methionyl-L-leucyl-L-phenylalanine/cytochalasin B (fMLP/CB). It also inhibited fMLP/CB-induced elastase release [118]. Chemotherapy with docetaxel (60 mg/m^2^) and carboplatin dosed every 3 weeks for 4 cycles to an ovarian carcinoma patient (66-year-old woman) was found to mask RA [119].

Andrographolide (25 *μ*M, for 16 h) in LPS-stimulated neutrophils accelerated apoptosis and inhibited autophagy-dependent extracellular TRAPs formation [77]. It also reduced neutrophil infiltration and NETosis in the ankle joints and relieved the systematic inflammation in adjuvant-induced arthritis C57BL/6 mice. Tanshinone IIA inhibited the NET formation of neutrophils in adjuvant-induced arthritis in female C57BL/6 mice [92]. On the other hand, triptolide (0.01–10 *μ*M) reduced viability and proliferation and induced apoptosis of RSF in a concentration-dependent manner in FLS from RA patients [79]. It also upregulated caspase-3 activity in the test system, whereas retinoic acid-platinum (II) complex (0.25–12 *μ*M) in MH7A cells induced apoptosis and caused the arrest of the cell cycle [90].

### 4.5. Improvement of Physiological Functions in RA Animals

Andrographolide (50 mg/kg/day) combined with methotrexate (2 mg/kg/week) for 35 days (injection) in complete Freund's adjuvant-induced arthritis in Wistar rats improved the serum marker. This may be attributed to the antioxidant activity of this compound, as evidenced by histological alterations in the liver [76]. Andrographolide when combined with methotrexate in complete Freund's adjuvant-induced arthritis in Wistar rats strengthened the antiarthritic capacity of methotrexate, reduced the inflammatory symptoms in animals, showed hepatoprotective activity, and significantly reduced serum TNF-*α*, IL-6, and IL-1*β* levels [76].

Triptolide loaded by a poly-*γ*-glutamic acid-grafted l-phenylalanine ethyl ester copolymer at 6.25–200 nM reduced the damaging effects on the liver, kidney, and spleen of mice [85]. In addition, triptolide (100 *μ*g/kg, i.p., 21 days) improved clinical arthritic conditions and joint destruction in collagen-induced arthritis in male DBA/1 mice [83], whereas triptolide-loaded poly(D,L-lactic acid) nanoparticles (0.05–0.2 mg/kg, p.o., for 14 days) in complete Freund's adjuvant-induced arthritis in male Wistar rats significantly inhibited arthritis and exerted a preferable anti-inflammatory effect with long-time administration [86].

In another study, triptolide loaded with miR-30-5p inhibitor significantly inhibited RA synovial fibroblast proliferation and increased apoptosis in collagen-induced arthritis female Sprague-Dawley rats [84]. This nanopreparation also downregulated immune system activation in rats.

Phytol (acyclic diterpene alcohol derived from chlorophyll) at 200 *μ*L (injection in the tail, for 10 days) was found to restore the oxidative-burst effect and induce a strikingly similar IFN-*β*-dependent pathway in DA rats [95].

Researchers suggested that it may be effective against naturally occurring genetic polymorphisms in the Ncf-1 gene that modulated the activity of the NADPH oxidase complex, which is potentially regulated in the severity of arthritis, whereas 11-epi-sinulariolide acetate significantly inhibited RA characteristics in adjuvant-induced arthritis in female Lewis rats [89].

Resiniferatoxin (10 *μ*L of 0.001 or 0.003%, injection) significantly improved arthritis with monoarticular inflammatory arthritis in evoked pain scores in arthritic male C57BL6 mice [96]. Similarly, tanshinone IIA (30 mg/kg, i.p., for 30 days) alleviated cartilage erosion and neutrophil infiltration in the ankle joints and reduced proinflammatory cytokine expression levels in sera in adjuvant-induced arthritis in female C57BL/6 mice [92]. In the complete Freund's adjuvant-induced arthritis rat model, phlomisoside F (5, 10, and 20 mg/kg, p.o., for 28 days) markedly offset the body weight loss, inhibited the paw edema, and reduced the arthritis scores and indices of the thymus and spleen [92]. Leflunomide (20 mg once daily) in combination with methotrexate improved signs, symptoms, and physical function in RA patients [91], while ginkgolide B (10, 20, and 40 *μ*M, i.p., for 43 days) in collagen II-induced arthritis male DBA/1J mice significantly decreased arthritis scores, synovial hyperplasia, and cartilage and bone destruction [88].

**Figure 4:**
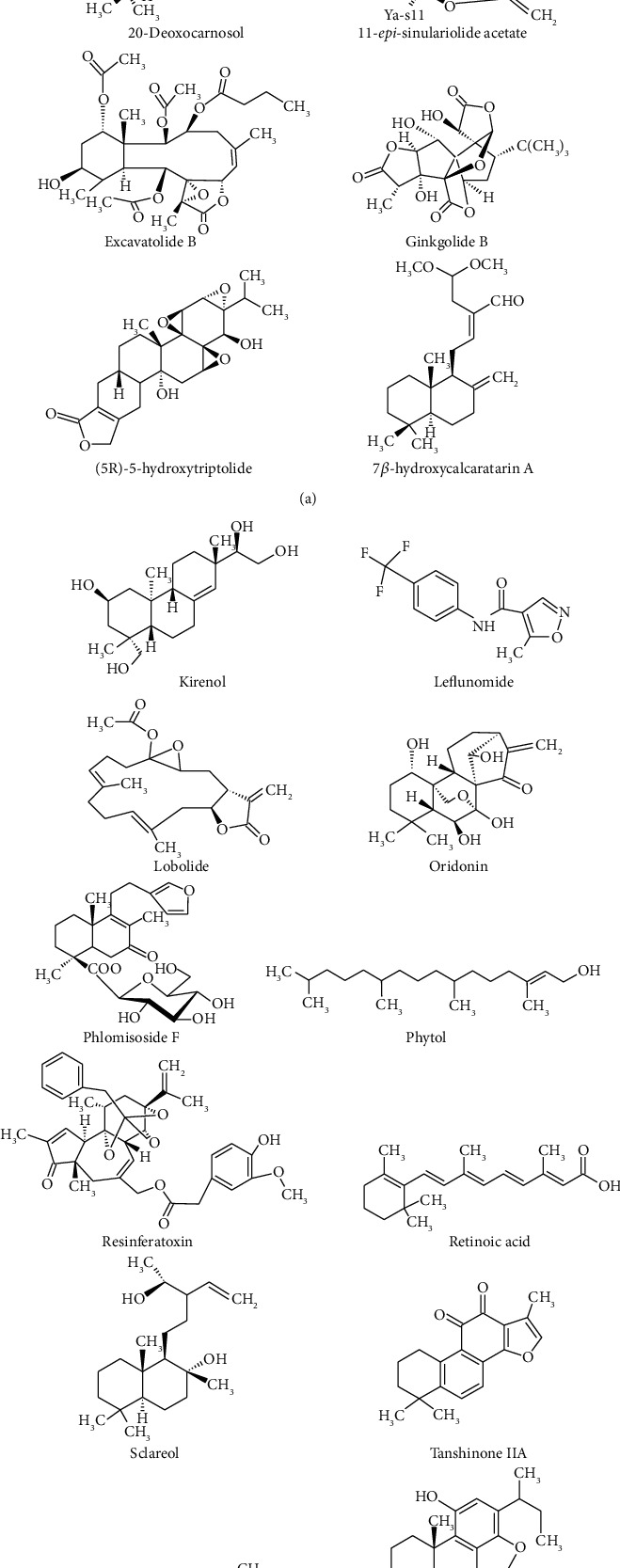
Chemical structures of some important anti-RA diterpenes and their derivatives.

The chemical structures of some anti-RA diterpenes and their derivatives are shown in Figure 4.

## 5. Discussion

Diterpenes and their derivatives are gaining popularity due to their intriguing biological and pharmacological properties. Thus far, hundreds of natural diterpene compounds from terrestrial and marine species have been described. Many of these compounds have become clinically effective.

Plants are an important source of diterpenes. Diterpenes can be linear, bicyclic, tricyclic, tetracyclic, pentacyclic, or macrocyclic. They are typically found in nature in a polyoxygenated form with keto and hydroxyl groups, which are frequently esterified by small-sized aliphatic or aromatic acids. For example, the anticancer drug taxol is used as a promising anticancer agent for ovarian, breast, and lung cancers. In addition, many of its synthetic derivatives are also examples of medicinal agents in the management of various diseases in humans. Docetaxel, sold under the brand name Taxotere®, is a taxoid antineoplastic drug used to treat a variety of malignancies, including locally advanced or metastatic breast cancer, metastatic prostate cancer, gastric adenocarcinoma, and head and neck cancer. Moreover, carboplatin, when combined with this drug, was found to mask RA in an ovarian carcinoma patient [119]. Similarly, ginkgolides are other promising diterpenes that have strong and specific antagonistic action against platelet-activating factors rising in shock, burns, ulceration, and inflammatory skin disorders [120]. Additionally, ginkgolide B exhibits multiedge-like anti-RA effects in in vitro and in vivo test models [88]. Meanwhile, the anti-RA diterpene resiniferatoxin (an ultrapotent vanilloid derived from the latex of *Euphorbia resinifera*) is promising for bladder hyperreflexia and diabetic neuropathy [120]. In short, diterpenes, diterpenoids, and their derivatives might be promising tools to manage RA and its consequences.

According to current knowledge [50], the most promising therapeutic targets in RA include the following:

1. Cytokines: TNF, IL-1, IL-1R, IL-6A, IL-6R, IL-2, IL-10, IL-15, IL-17, IL-17R, IL-18, and IFN-*γ*
2. Chemokines: CCL2, CCR9, CX3CL1, CCR1, CCR2, CCR5, CCR7, CXCL10, CXCL12, CXCL13, CXCL16, CXCR1/2, CXCR3, CXCR4, and CXCR7
3. Other related proteins: BTK, CD3, CD11a, CD19, CD20, CD80, GRK2, GM-CSF, IL-23, IRAK-4, JAK, MEK, MMP-9, p38 MAPK, and TLR-4
4. Small molecular metabolites: PGD2, PGE2, PGI2, PGJ2, PGF2*α*, TXA2, LTB4R, CysLT1R, ALX, PAFR, ROS, iNOS, CB2, and FFAH

This review suggests that diterpenes and their derivatives act on the cytokines (IL-1, IL-1*α*, IL-1*β*, IL-6, IL-8, IL-10, IL-17, IL-17A, IL-21, IFN-*γ*, TNF-*α*, TGF-*β*1, MMPS (e.g., MMP-1, MMP-2, MMP-3, MMP-9, and MMP-13), and MCP-1), chemokines (CCl5 and CXCL10), and many proteins (IL-23, p38 MAPK, ERK, NF-*κ*B, TRAP, cathepsin K, CD11b, PPAR-*γ*, VEGF, VEGFR, Ang-1, Ang-2, Tie2, JNK, RANK/RANKL, OPG, p-I*κ*B, TREM-1, JAK2, STAT3, iNOS, COX-2, PI3K/AKT, lncRNA GAS5, 5-LOX, WAKMAR2/miR-4478/E2F1/p53, ADAMTS-4, and PGE2).

It appears that diterpenes and their derivatives have multiedge-like actions on different RA models. These compounds exerted anti-RA effects through the cytokine, chemokine, inflammatory/noninflammatory proteins, and small molecular metabolites pathways. Among the diterpenes, triptolide and its derivative (5R)-5-hydroxytriptolide have been found to display promising anti-RA activity in various test systems.

Other hopeful anti-RA diterpenes and diterpenoids found in this updated review include carnosol and carnosic acid and their derivatives, excavatolide B, Kirenol, ginkgolide B, 11-epi-sinulariolide acetate, oridonin, phlomisoside F, phytol, retinoic acid, resiniferatoxin, sclareol, and xylopic acid among others.

A novel triptolide derivative (also known as LLDT-8), which exhibited anti-RA therapeutic properties, is currently in phase II clinical studies in China [63]. Diterpenes and their derivatives act through multidimensional pathways in different RA animal models. Moreover, triptolide-loaded nanocomplexes also improved anti-RA potential in experimental modalities. Besides these compounds/formulations, andrographolide, tanshinone IIA, and its derived compound cryptotanshinone also displayed promising anti-RA effects in test systems.

## 6. Conclusion

To date, many natural products that have the anti-RA capacity, including those obtained from medicinal plants and marine organisms, have been identified. The sources of diterpenes and diterpenoids are widely distributed. Natural products, including medicinal plant-derived chemicals, are a prominent source of semisynthetic and synthetic derivatives. Hence, nature and modern medicinal science are capable of providing new and more effective diterpene derivatives. Diterpenes and their derivatives have been shown to possess promising immunomodulatory properties in various experimental models; therefore, these natural bioactive compounds are a promising adjuvant pharmacotherapy in RA.

## Figures and Tables

**Table 1:** Diterpenes, diterpenoids, and their derivatives in various rheumatoid arthritis models.

Diterpenes/derivatives	Concentration/dose test system	Results/mechanisms	References
Sclareol	3.125–100/3.125–12.5 *μ*M SW982 human synovial cell lines in vitro	↓TNF-*α*, ↓IL-6, ↓NF-*κ*B ↓p38, ↓MAPK ↓ERK	[74]
	5–10 mg/kg (i.p., every other day over 21 days) collagen-induced arthritis DBA/1J mice in vivo	↓Swelling in paws, ↓serum anti-CII antibodies ↓IL-1*β*, ↓IL-6, ↓TNF-*α*, ↓IL-17 ↓Th17, ↓Th1	
Excavatolide B	10 *μ*M LPS-stimulated RAW 264.7 cells in vitro	↓Multinucleated cell ↓Actin ring formation ↓TRAP, ↓MMP-9, ↓K	[75]
	2.5–5 mg/kg (s.c.) type II collagen-induced arthritis in rats in vivo	↓RA characteristics, ↑histopathological features ↓TRAP-positive multinucleated cells ↓Cathepsin K, ↓MMP-2, ↓MMP-9, ↓CD11b ↓NFATc1, ↓IL-17A, ↓CSF	
Andrographolide	2.5–10 *μ*M bone marrow macrophages (BMM) cells in vitro 5–30 mg/kg (i.p., every other day for 8 days) C57/BL6 mice in vivo	↓RANKL ↓NF-*κ*B ↓ERK/MAPK osteoclastogenesis ↓bone loss	[70]
	50 mg/kg/d combined with methotrexate (2 mg/kg/week, i.p. for 35 days) Freund's adjuvant-induced arthritis in Wistar rats in vivo	↑Methotrexate effect hepatoprotective ↓TNF-*α*, ↓IL-6, ↓IL-1*β*	[76]
	25 *μ*M LPS-stimulated neutrophils in vitro	↑Apoptosis ↓TRAPs	[77]
	25–50 mg/kg (i.p., for 37 days) adjuvant-induced arthritis C57BL/6 mice in vivo	↓Neutrophil infiltration ↓NETs in the ankle joints ↓Systemic inflammation	
Triptolide	0.1 mg/kg (p.o., for 28 days) type II collagen-induced arthritis in rats in vivo	Delayed onset of arthritis ↓Arthritis incidence ↓Clinical arthritis severity score ↓Histopathological arthritis severity score ↓Cell-mediated immunity	[78]
	0.01–10 *μ*M RSF cells in vitro	↓Viability, ↓proliferation, ↑apoptosis ↑Caspase-3 ↓PPAR-*γ* ↑DNA fragmentation	[79]
	SW1353 cells synovial fibroblasts chondrocytes in vitro	↓MMP-3, MMP-13 ↓IL-1, -17 ↓TNF-*α*	[80]
	11–45 *μ*g/kg/day (i.g., for 28 days) bovine type II collagen-induced arthritis DA rats in vivo HFLS-R cells HUVEC cells in vitro	↓Arthritis scores ↓Density of capillaries, small, medium, and large vessels in the synovial membrane tissues of inflammatory joints ↓Matrigel-induced cell adhesion ↓VEGF, ↓VEGFR, ↓Ang-1, ↓Ang-2, ↓Tie2 ↓TNF-*α*, ↓IL-17, ↓IL-1*β*, ↓p38, ↓JNK	[81]
	1–4 nM/L MDA-MB-23 human breast tumor cells U266 multiple myeloma cells PC-3 prostate tumor cells in vitro	↓RANKL ↓NF-*κ*B ↓Osteoclastogenesis	[72]
	6.25–200 nM LPS-stimulated U937 cells in vitro	↓TREM-1 ↓JAK2, ↓STAT3 ↓TNF-*α*, ↓IL-1*β*, -6	[82]
	9.31–18.62 *μ*g/kg (p.o., for 21 days) collagen-induced arthritis rats in vivo	↓TREM-1/DAP12 ↓JAK2, ↓STAT3 ↓TNF-*α*, ↓ IL-1*β*, ↓IL-6	
	2.5–40 nM bone marrow macrophages in vitro male C57BL/6 mice in vivo	↓Osteoclasts development ↓Bone resorption ↑IL-10 ↑TGF-*β*1	[73]
	10, 30, and 50 nM HFLS-RA cells in vitro	↓TNF-*α* ↓JNK ↓Migration	[83]
	100 *μ*g/kg (i.p., 21 days) collagen-induced arthritis in DBA/1 mice *in vivo*	↑Clinical arthritic conditions ↓Joint destruction	
Triptolide (TP) loaded with miR-30-5p inhibitor (MSNs@PCM@TP)	TP 15 mg loaded in nanoformulation. TP 50 *μ*g/kg (i.p.) MSNs@PCM@TP 100 *μ*g/mL (i.p.) collagen-induced arthritis in rats in vivo RA FLS cells in vitro	↓Proliferation ↑Apoptosis ↓Immune system activation in rats	[84]
Triptolide loaded by a poly-*γ*-glutamic acid-grafted l-phenylalanine ethyl ester copolymer	6.25–200 nM RAW264.7 cells in vitro, 0.5–2 mg/kg (i.p.) mice in vivo	Anti-RA effect ↓Triptolide toxicity on the liver, kidney, and spleen	[85]
Triptolide-loaded poly (d,l-lactic acid) nanoparticles	0.05–0.2 mg/kg (p.o., for 14 days) induced arthritis in rats in vivo	↓Arthritis anti-inflammatory	[86]
(5R)-5-Hydroxytriptolide	12.5–50 nM peripheral blood and synovial fluid of RA patients in vitro	↑OPG, ↑OPG/RANKL ↓IL-1*β*, ↓IL-6, ↓IL-21, ↓IL-23 ↑IL-10 ↓p-I*κ*B	[69]
	Murine RAW264.7 cells in vitro	↓TRAP-positive cells	
	100 nM/mL genome-wide microarray assay in RA patients	Influenced the FLS especially in the process of immune-related pathways	[60]
	50–100 nM RA FLS cells in vitro	↓MMP-3, ↓IL-1, ↓IL-6, ↓WAKMAR2/miR-4478/E2F1/p53	[63]
Kirenol	100–200 *μ*g/mL RA FLS cells in vitro	↓IL-6, ↓migration, ↓invasion	[87]
	7.5–30 mg/kg (i.p., for 21 days) collagen-induced arthritis DBA/1 mice in vivo	↓IL-6, ↓synovium hyperplasia, ↓cartilage erosion	
Ginkgolide B	10, 20, 40 *μ*M (i.p., for 43 days) collagen II-induced arthritis male DBA/1J mice in vivo	↓Arthritis scores, ↓synovial hyperplasia ↓Cartilage and bone destruction ↓IL-1*β*, ↓IL-6, ↓MCP-1, ↓TNF-*α*, ↓MMP-3, ↑IL-10	[88]
	5–80 *μ*M LPS-induced FLS cells in vitro	↓Viability ↓Caspase-3, ↓Bax, ↓Bcl-2 ↓MMP, ↓Wnt5a, ↓JNK, ↓p65	
11-epi-Sinulariolide acetate	1, 10, 25, 50 *μ*M LPS-stimulated murine macrophages in vitro	↓iNOS ↓COX-2	[89]
	9 mg/kg (s.c., once every 2 days from day 7 to day 28 postimmunization) adjuvant-induced arthritis in Lewis rats in vivo	↓RA characteristics ↓Cathepsin K ↓MMP-9, ↓TRAP ↓TNF-*α*	
Retinoic acid-platinum (II) complex	0.25–12 *μ*M MH7A cells in vitro	↓TNF-*α* ↑Apoptosis, ↑cell cycle arrest ↓MEK/NF-*κ*B	[90]
	2 and 5 mg/kg (i.g.) Sprague-Dawley rats in vivo	↓IL-1*β*, ↓IL-6, ↓IL-8, ↓MMP-1, ↓MMP-13 ↓iNOS, ↓COX-2 mRNA	
Leflunomide in combination with methotrexate	20 mg once daily in RA patients	↓RA signs and symptoms improved physical function	[91]
Oridonin	2–10 *μ*g/mL, 24–72 h RA HFLS cells in vitro	↓Proliferation ↑Bax, ↓caspase-3, ↓IL-1*β* ↓GFP-LC3 punctate dots, ↓ATG5, ↓Beclin1	[52]
Tanshinone IIA	30 mg/kg (i.p., for 30 days) adjuvant-induced arthritis C57BL/6 mice in vivo	↓Proinflammatory cytokines ↓Cartilage degradation, ↓neutrophils infiltration ↓IL-6, ↓TNF-*α*, ↓neutrophil NETosis	[94]
	1–80 *μ*M RA HFLS cells in vitro	↑Cytotoxicity, ↑apoptosis ↑lncRNA GAS5, ↑caspase-3, ↑caspase-9 ↓PI3K/AKT	[93]
Phlomisoside F	5, 10, 20 mg/kg (p.o., for 28 days) adjuvant-induced arthritis Wistar rats in vivo	Markedly offset the bodyweight loss, ↓paw edema, ↓arthritis scores ↓TNF-*α*, ↓IL-1*β*, ↓IL-6, ↓COX-2, ↓5-LOX, ↑IL-10	[92]
Phytol	200 *μ*L (injection in tail, for 10 days) DA rats in vivo	Restored oxidative-burst effect induced a strikingly similar IFN-*β*-dependent pathway. effective against naturally occurring genetic polymorphisms in the Ncf-1 gene that modulate the activity of the NADPH oxidase complex, which is potentially regulated in the severity of arthritis.	[95]
Resiniferatoxin	10 *μ*L of 0.001–0.003% (injection) evoked pain scores arthritic C57BL6 mice in vivo	↓Arthritis ↓Inflammation	[96]
Xylopic acid nanoformulation	200 *μ*g/mL RAW 264.7 cells in vitro, 5 mg/kg (i.v.) adjuvant-induced arthritis in SD rats in vivo	Anti-inflammatory Antirheumatoid	[97]
Cryptotanshinone	6–18 mg/kg (p.o., for 16 days) type II collagen-induced arthritis in Wistar rats in vivo	↓NF-*κ*B ↓IkB-*α*	[71]
	5–20 *μ*M LPS-induced Raw264.7 macrophages in vitro		
	20–60 mg/kg (p.o., for 6 weeks) adjuvant-induced arthritis in DBA/1 mice in vivo	↓Inflammation and joint destruction ↓p300 ↓STAT3	[98]
Carnosic acid	30–60 mg/kg (i.p., 4 weeks) collagen-induced arthritis in C57BL/KsJ-db/db mice in vivo, 10 or 20 *μ*M mouse bone marrow cells in vitro	↓Osteoclasts ↓Bone loss ↓Inflammation ↓ROS ↓p38	[99]

↑, increase; ↓, decrease; CSF, macrophage colony-stimulating factor; NETs, neutrophil extracellular traps; HFLS-RA, human fibroblast-like synoviocytes of rheumatoid arthritis; HUVECs, human umbilical vein endothelial cells; TREM-1, triggering receptor expressed on myeloid cells-1; ROS, reactive oxygen species; JAK, Janus kinase; STAT3, signal transducer and activator of transcription 3; RSF, rheumatoid synovial fibroblasts; BMM, bone marrow macrophages; JNK, c-Jun N-terminal kinase; RSF, rheumatoid synovial fibroblasts; RANKL, receptor activator of NF-jB ligand; OPG, osteoprotegerin; IL, interleukin; WAKMAR 2, wound and keratinocyte migration–associated long noncoding RNA 2; NF-*κ*B, nuclear factor-*κ*B; TRAP, tartrate-resistant acid phosphatase; lncRNAs, long noncoding RNAs; GAS5, growth arrest-specific 5; I*κ*B*α*, nuclear factor of kappa light polypeptide gene enhancer in B cells inhibitor, alpha.

**Table 2:** Diterpenes and their derivatives targeting other proteins in rheumatoid arthritis.

Target proteins	Diterpenes/diterpenoids or their derivatives	Reference
IL-23	(5R)-5-Hydroxytriptolide	[69]
p38 MAPK	Sclareol	[74]
ERK	Triptolide, andrographolide, sclareol	[70, 74, 81]
NF-*κ*B	Lobolide, andrographolide, carnosol, sclareol, (5R)-5-hydroxytriptolide, retinoic acid-platinum (II) complex	[60, 70, 74, 90, 112, 116]
TRAP	11-epi-Sinulariolide acetate, (5R)-5-hydroxytriptolide, excavatolide B, andrographolide	[69, 75, 89, 115]
MMPs	Triptolide, 11-epi-sinulariolide acetate, excavatolide B, ginkgolide B, retinoic acid-platinum (II) complex	[75, 80, 88–90]
Cathepsin K	11-epi-Sinulariolide acetate, excavatolide B	[75, 89]
CD11b	Excavatolide B	[75]
PPAR-*γ*	Triptolide	[79]
VEGF, VEGFR, Ang-1, Ang-2, Tie2	Triptolide	[81]
JNK	Triptolide, ginkgolide B	[81, 83, 88]
RANK/RANKL, OPG	Triptolide, andrographolide, (5R)-5-hydroxytriptolide	[69, 70, 72, 113, 114]
p-I*κ*B	(5R)-5-Hydroxytriptolide	[69]
TREM-1	Triptolide	[82]
JAK2		
STAT3	Triptolide, cryptotanshinone	[82, 98]
iNOS	Sclareol, 11-epi-sinulariolide acetate, carnosol, carnosic acid, carnosic acid-12-methylether, 20-deoxocarnosol and abieta-8,11,13-triene-11,12,20-triol, and retinoic acid-platinum (II) complex, aphamines A–C	[89, 90, 102, 103, 110, 112]
COX-2	Sclareol, 11-epi-sinulariolide acetate, retinoic acid-platinum (II) complex	[89, 90, 102, 110]
PI3K/AKT	Tanshinone IIA	[93]
lncRNA GAS5		
5-LOX	Phlomisoside F	[92]
WAKMAR2/miR-4478/E2F1/p53	(5R)-5-Hydroxytriptolide	[63]
ADAMTS-4	Triptolide, carnosol, carnosic acid, carnosic acid-12-methylether, 20-deoxocarnosol, abieta-8,11,13-triene-11,12,20-triol	[80, 112]
PGE2	Carnosol, carnosic acid, carnosic acid-12-methylether, 20-deoxocarnosol, abieta-8,11,13-triene-11,12,20-triol	[112]

## Data Availability

The data used to support the findings of this study are available from the corresponding author upon request and are cited within the article as references.
